# The Treatment of Facial Lupus Vulgaris

**Published:** 1907-07-13

**Authors:** J. Goodwin Tomkinson

**Affiliations:** Assistant Medical Electrician, Glasgow Western Infirmary


### Dermatology.

### THE TREATMENT OF FACIAL LUPUS VULGARIS.

By J. GOODWIN TOMKINSON, M.D., Assistant Medical Electrician, Glasgow Western Infirmary.

Of the many skin diseases more or less amenable to electrotherapeutics, cutaneous tuberculosis is prominent by reason of its frequency. A short account of the method which I employ in hospital practice for the treatment of facial Lupus vulgaris may be of interest to those possessing an *x*-ray apparatus.

While fully convinced of the high value of the treatment introduced by Finsen—especially after hearing the admirable paper read at the Paris Congress on tuberculosis in 1905 by my teacher and friend, De Beurrman—I find that many cases yield with far greater rapidity to a combination of *x*-ray treatment with mildly caustic methods, and the results of this combined method have determined me to abandon, if not entirely, yet very largely, the light treatment of Lupus vulgaris. Many cases with which one is confronted in hospital practice are so extensive that their treatment by the Finsen method would be virtually interminable. And hence the points that the *x*-rays are more expeditious, are applicable to wider areas of disease, give a resulting cicatrix of high æsthetic value, and have a tendency to reduce any keloidal condition already existing—primary or the effect of previous treatment—indicate the exhibition of this remedy.

### Surgical Interference.

In facial lupus vulgaris permit me to dismiss the question of surgical intervention as altogether contraindicated, save in cases of extremely limited dimensions. At the same time it would be only just to recall the excellent results obtained, especially by exponents of the French School of Dermatology, by multiple scarification, particularly when combined with the application of 5 per cent. aqueous solution of permanganate of potash as recommended by Hallopeau, and practised by him at L'Hôpital Saint Louis, Paris. Occasionally the sharp spoon is of value, but in my own work at the Glasgow Western Infirmary, in so far as the treatment of facial lesions is concerned, I use no local adjunct to *x*-ray therapy more drastic than the caustic applications about to be described.

### How to Start the Treatment.

At the outset of the treatment of a case the crusts —if any exist—are first removed, and these are usually quickly detached by the application of a salicylic ointment. A tentative *x*-ray exposure of from three to five minutes is afterwards made, directed upon a small area of the lesion; after three or four days the exposure is repeated for five minutes daily, unless contraindicated, until the whole lesion has been treated some three or four times. The lesion is then covered with a strong salicylic acid plaster, and for this purpose Unna's 50 per cent. salicylic acid and creosote pflastermulle is employed. The plaster is renewed daily, preceded if badly borne by the use of 10-20 per cent. aqueous solution of cocaine. When the tuberculous nodules are in the main removed the lesion is swabbed with cocaine solution, then dried, and painted with the following preparation:

| | | |
|---|---|---|
| ℞ | Acid. carbolici ... ... ... | 50 per cent. |
| | Acid. lactici ... ... ... | 15 ,, |
| | Acid. salicylici ... ... ... | 15 ,, |
| | Alcohol. absolut. ... ... ... | 20 ,, |

(Well agitated before application, as there is a considerable sediment.) A few minutes afterwards the lesion is painted with the following solution:

| | | |
|---|---|---|
| ℞ | Acid. carbolici ... ... ... | 80 per cent. |
| | Alcohol. absolut. ... ... ... | 20 ,, |
| | | (Billet) |

(This second formula is that employed in Billet's phenol treatment.)

The lesion is then dressed with sterilised lint and carbolic oil (1-30) for a day or two, and thereafter with 20 per cent. aqueous ichthyol solution, until healing—often relatively rapid—has taken place: in some instances the application of the ichthyol solution is omitted until the lesion has been replastered and re-cauterised perhaps three or four times. After healing by the use of sterilised lint and 20 per cent. aqueous ichthyol solution—which may be accelerated by first bathing the part with a little of the solution to which 25-50 per cent. methylated spirit has been added (cocaine swabbing is indicated)—the treatment by *x*-rays is recommenced; five to ten minutes, usually the former, is the length of time of exposure. After this second period of *x*-ray exposures, the duration of which is determined by the individual case, the lesion is again "broken down" with the salicylic acid pflastermulle and cauterised with the "tri-acid" and carbolic solutions. After the surface has again been healed with ichthyol solution the affected area is submitted to a few *x*-ray exposures, and the patient is sent home if residing in the country, or, if living in town, to a convalescent home if possible. During the cessation of the more vigorous treatment the cicatrix in some instances is treated by mercurial inunction or the application of ½ per cent. perchloride of mercury lotion. The duration of this course obviously varies with the extent of the lesion and the idiosyncrasy of the patient, but, in most cases, at the end of three or four months' treatment is discontinued for a long period, during which the patient returns occasionally for inspection, and continued improvement is often recorded. If necessary, as is usually the case, the treatment is repeated once or more.

Whenever constitutional treatment is indicated it should be instituted: the medicinal agents most frequently employed are syr. ferri iodidi, syr. Easton, ol. morrhuæ, and saline aperients; a generous dietary and residence amidst hygienic surroundings are also enjoined.

